# Effect of TGF-β/smad signaling pathway blocking on expression profiles of miR-335, miR-150, miR-194, miR-27a, and miR-199a of hepatic stellate cells (HSCs) 

**Published:** 2017

**Authors:** Parivash Davoodian, Mehrdad Ravanshad, Seyed Younes Hosseini, Sayyad Khanizadeh, Mohammad Almasian, Azim Nejati Zadeh, Hamed Esmaiili Lashgarian

**Affiliations:** 1 *Infectious and Tropical Diseases Research Center, Hormozgan University of Medical Sciences, Bandar Abbas, Iran*; 2 *Department of Virology, Faculty of Medical Sciences, Tarbiat Modares University, Tehran, Iran*; 3 *Department of Bacteriology and Virology, Shiraz University of Medical Sciences, Shiraz, Iran*; 4 *Hepatitis Research Center, Lorestan University of Medical Sciences, Khorramabad, Iran*; 5 *School of medicine, Lorestan University of Medical Sciences, Khorramabad, Iran*; 6 *Research Center for Molecular Medicine, Hormozgan University of Medical Sciences, Bandar Abbas, Iran*

**Keywords:** Fibrosis, microRNAs, HSCs, TGF-β, shRNA

## Abstract

**Aim:**

The aim of this study was to determine the effect of inhibition of TGF-β/smad signaling on the expression profiles of miR-335, miR-150, miR-194, miR-27a, miR-199a of hepatic stellate cells (HSCs).

**Background:**

Liver fibrosis is excessive deposition of extracellular matrix proteins due to ongoing inflammation and HSC activation that occurs in most types of chronic liver diseases. Recent studies have shown the importance of microRNAs in the pathogenesis of chronic liver diseases.

**Methods:**

In this study, for inhibition of TGF-β smad-signaling pathway, expressing Smad4 shRNA plasmids were transfected into HSCs. Subsequently, using Real Time-PCR, we measured the expression levels of miR-335, miR-150, miR-194, miR-27a and miR-199a.

**Results:**

Gene expression analysis showed that downregulation of Smad4 by vector Smad4shRNA significantly increased the expression levels of miR-335 (P<0.01) and miR-150 (P<0.001) and decreased the expression level of miR-27a (P<0.05).

**Conclusion:**

The results of this study suggest that blocking TGF-β smad-signaling can also differentially modulate microRNA expression in support of activation and fibrogenesis of HSCs.

## Introduction

 Hepatic fibrosis results from a wound healing response against chronic damage to the liver. The main causes of liver fibrosis are chronic infection with hepatitis viruses, non-alcoholic steatohepatitis (NASH) and alcohol abuse. Excessive accumulation of extracellular matrix proteins (ECM) during chronic damage leads to destruction of hepatic architecture and formation of fibrous scar. As a result, liver fibrosis can progress toward cirrhosis and hepatocellular carcinoma (1).

There are several types of cell associated with the progress of liver fibrosis, among which hepatic stellate cells (HSCs) are very influential. These cells are considered as the most important ECM-producing cells following liver damage (2). During chronic liver injury, HSCs undergoes phenotype activation in such a way that produce fibrotic markers se well as extracellular proteins (3). A large number of studies have shown that activation of the TGF-β/Smad signaling pathway strongly contributes to triggering the fibrogenesis phenomenon in HSCs (4-6).

**Table 1:** miRNA reverse transcription (RT) - qPCR primers

miRNA name	Primer sequence 5ʹ → 3ʹ
hsa-miR-27a	RT: GTCGTATCCAGTGCAGGGTCCGAGGTATTCGCACTGGATACGACTGCTCA
	F: AGAGGGCTTAGCTGCTTGT
hsa-miR-194	RT: GTCGTATCCAGTGCAGGGTCCGAGGTATTCGCACTGGATACGACTCCACA
	F: GGTGTAACAGCAACTCCATGT
hsa-miR-199a	RT: GTCGTATCCAGTGCAGGGTCCGAGGTATTCGCACTGGATACGACGAACAG
	F: CCCAGTGTTCAGACTACCTGT
hsa-miR-150	RT: GTCGTATCCAGTGCAGGGTCCGAGGTATTCGCACTGGATACGACCACTGG
	F: CACAGTCTCCCAACCCTTGT
hsa-miR-335	RT: GTCGTATCCAGTGCAGGGTCCGAGGTATTCGCACTGGATACGACACATTT
	F: GCAGGTCAAGAGCAATAACGA

Universal reverse primer: GTGCAGGGTCCGAGGT, miRNA complementary specific sequences are underlined

MicroRNAs are small noncoding RNAs molecules that regulate biological functions such as apoptosis, proliferation, physiological and pathophysiological processes and perform their action posttranscriptionally through base pairing with target messenger RNA (4). Recent studies suggest that microRNAs expression profile contributes to progress of fibrosis (4). For example, it has been demonstrated that miR-29 regulates liver fibrosis through TGF-β/Smad signaling pathway in HSCs. Hence, the expression of the miR-29 family is considerably down-regulated in livers of patients with progressive fibrosis. On the other hand, overexpression of miR-29 molecule has been demonstrated to alleviate the fibrosis progression (5, 6). Similar reports considering fibrotic, anti-fibrotic and anti-migratory roles for miRNAs that they can contributed to liver fibrosis. Some miRNAs including miR-335, miR-150 and miR-194 have been indicated as HSCs modulatory molecules while others like miR-27a and miR-199a are accepted as HSC activators during fibrogenesis (5). However, their role in fibrogenesis remains to be definitely investigated and their link to TGF-β/Smad signaling also needs further assessment.

The aim of the present study was to investigate the effect of TGF-β/Smad pathway blocking on the expression profile of miR-335, miR-150, miR-194, miR-27a and, miR-199a in an activated HSCs cell

## Methods

**Cell culture and activation of hepatic stellate cells (HSCs)**

The human hepatic stellate cell line LX-2 (an immortalized human stellate cell line) was kindly provided by Dr. Scott L. Friedman (Mount Sinai School of Medicine, New York, NY, USA) (7). The cells were cultured in Dulbecco’s modified Eagle’s medium (DMEM) complete medium. Furthermore, in order to activate cells, they were treated by TGF-b containing medium as described before (8). 

**Transient transfection of hepatic stellate cells**

To investigate the effect of TGF-smad blocking on miRNA profile, different groups of cells were transfected by Smad4 shRNA expressing plasmid vector. The activated LX-2 by TGF-b, normal LX-2 cell and transfected with empty plasmid as negative control constituted the experiment groups. Transfection was performed according to manufacturer’s instructions.

**Reverse transcription and real-time PCR for detection of miRNA expressions**

To evaluate miRNAs expression level, qRT-PCR was performed using stem loop primers (Table 1). The expression level of U6 snRNA was determined usnig the following primers: forward 5ʹ - CTCGCTTCGGCAGCACATATACT -3ʹ, reverse 5ʹ - ACGCTTCACGAATTTGCGTGTC -3ʹ, as used to normalize the fold change of miRNAs for different experiments (9). Following RNA extraction with the QIAzol reagent, the samples were converted to cDNA using Revert Aid First Strand cDNA Synthesis Kit (Thermo Fisher Scientific, USA) according to the following: 4 µL of extracted miRNA, was added to 1.5 µL stem-loop RT primer and 5 µL distilled water; subsequently 10.5 µL reactions were incubated in an ABI Thermo cycler (ABI Inc. USA) at 70°C for 15 min. The tubes were placed on ice and 4 µL 5x Buffer, 2µL dNTP (10 mM), 2µL DTT (10 mM), 1 µL RNase inhibitor (20U) and 1 µL reverse transcriptase enzyme (50U) were added. The cDNA synthesis was performed as follows: 42 °C for 40 min and at 72 °C for 5 min for inactivation of the reversecriptase enzyme, then cDNA products were kept at -20 °C until use. Following cDNA synthesis, real-time PCR was performed under the following conditions: 95°C for 30 s, 40 cycles of 95°C for 10 s, 60°C for 25 s and 72°C for 20 s, followed by 40°C for 20 min.

Amplification signals for changed samples were normalized to U6 snRNA signal, then delta-delta CT (2-^∆∆C^T) method was employed for comparing mRNA levels of tests versus control which was finally represented as abundance relative quantification.

**Statistical analysis**

Data were presented as bar graphs obtained from at least three independent experiments. Statistical analysis was performed using the Graph Pad Prism software version 6. The statistical significance level between controls and treated groups was assessed further by Tukey post-test and P < 0.05 was considered to indicate a statistically significant difference.

## Results

The results of previous studies have shown that Smad4 shRNA could significantly cause down-regulation of fibrotic genes: smad4, COL1A, α-SMA and TIMP1. It means that TGF-b stimulation can induce HSCs activation and blocking the pathway may subvert the activation mechanism.

**Figure 1:**
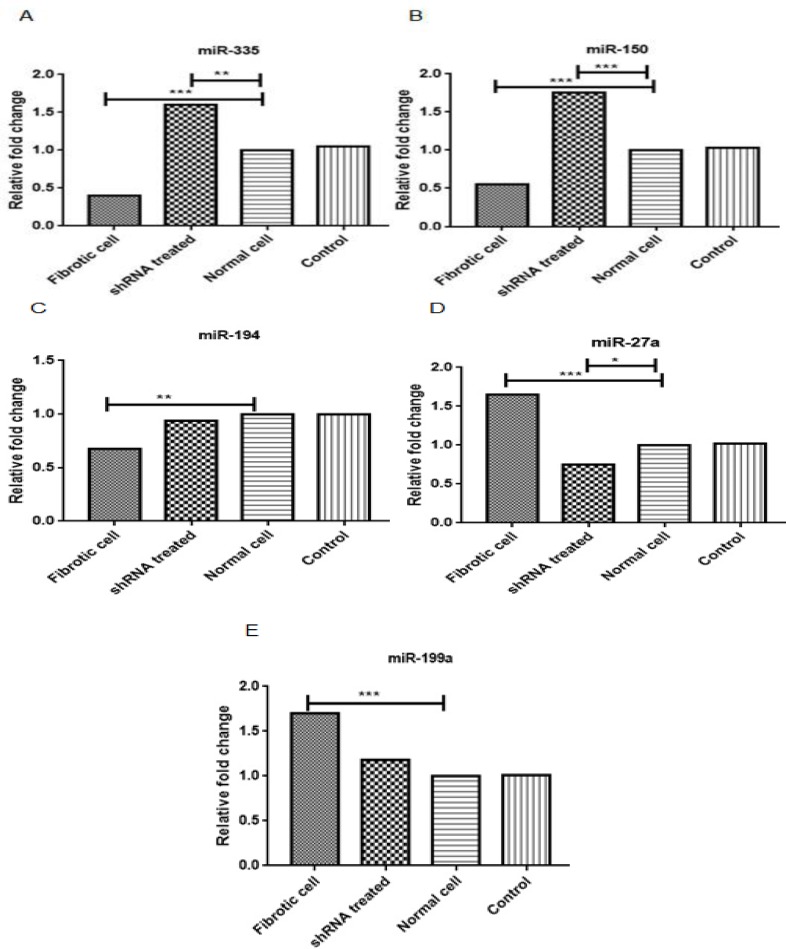
The results of Real Time-PCR on the expression level of miR-335, miR-150, miR-194, miR-27a, and miR-199a in the 4 groups, namely fibrotic cells as positive control group, shRNA treated as test group, normal and negative cells (treated with empty plasmid) as control group. (Fig 1A-E). *P <0.05, **P <0.01 and ***P <0.001 indicate the significant fold changes of test groups compared to empty GFP plasmid and normal LX-2 cells

Following the blocking of TGF-β/smad signaling pathway through knockdown of Smad4 by Smad4 shRNA(10), the expression profiles of miR-335, miR-150, miR-194, miR-27a, and miR-199a were detected using real time -PCR (figure 1). As mentioned above, the cells were divided in 4 groups, namely cells treated with shRNA, activated cells, untreated cells (normal cell) and cells treated with empty GFP plasmid as negative control group. It was observed that the expression of miR-150, miR-335 and miR-194 was significantly downregulated in comparison with untreated cells or normal cells and the negative control group (LX-2 cells transfected with empty plasmids expressing GFP), while the expression of miR-27a and miR-199a was significantly upregulated compared to control groups. The results of qPCR for treated groups showed that Smad4 shRNA caused significant downregulation of miR-27a and upregulation of miR-150 and miR-335, while the expression level of miR-199a and miR-194 did not show any significant difference (Fig 1A-E). This result indicates that Smad4 blocking could affect the expression profile of miR-27a, miR-150 and miR-335. 

## Discussion

The molecular functions of miRNAs have been well documented in regulation of fibrotic gene expressions in liver fibrosis. The fibrogenesis effects of HSCs have also been well documented (3,5). Although there is limited insight on the importance of miRNAs in liver tissue development and pathogenesis, these regulatory small oligonucleotides are likely to regulate differentiation and interaction with other cellular signaling pathways. Thus, increasing our understanding of the function of miRNAs in liver physiology will help us understand liver diseases such as liver fibrosis (11, 12). Utilization of short hairpin RNA (shRNA) and small interfering RNA (siRNA) to treat liver fibrosis and their silencing effects on the down-stream fibrotic genes of TGF-β1/smad signaling pathway have been well studied (13,14).

In this study, in order to find out the crosstalk between the microRNA machinery and TGF-β/smad signaling, we used smad4 shRNA to inhibit the TGF-β1 signaling pathway. Thereafter, we investigated the effect of blocking TGF-β/smad signaling pathway on the expression profile of five effective pro- and anti-fibrotic microRNAs in the HSCs. As mentioned above, our results show that disruption of TGF-β/smad signaling pathway by shRNA could decrease fibrogenesis through downregulation of the down-stream fibrotic gene expressions of α-sma, col1a and TIMP1(10). However, in addition to regular proteins such as col1a and TIMP1 that act in final steps of fibrogenesis, miRNAs may also play a role in establishment of fibrosis. In this study,to answer this question, whether blocking of the TGF-β pathway is involved in the modulation of effective miRNAs in liver fibrogenesis or no, the pattern of several miRNAs was underwent expression analysis. 

In our study, we observed that blocking the TGF-β/smad signaling pathway can modulate expression of miR-335, miR-150a and miR-27a, while we observed no significant effect on the expression of miR-199a and miR-194. The expression pattern of these microRNAs in liver diseases have been investigated in various studies (5) and the effect of miR-335, miR-150a and miR-27a on the liver fibrosis well documented. The experimental assays demonstrated that in the pathogenesis of liver fibrosis, miR-335; miR-150a and miR-194 have antifibrotic effects while miR-199a and miR-27a exhibit strong profibrotic properties (5). Venugopal et al. have shown that overexpression of miRNA-194 and miRNA-150 in LX-2 cells resulted in inhibition of rac1 and c-myb expression which consequently alleviated liver fibrosis and HSC activation process (15). Also, Chen et al. have demonstrated that miR-335 significantly decreases during activation of HSCs and liver fibrosis, and restoring the expression of miR-335 controls HSC migration and decrement of collagen type I expression. Previous reports discovered that tenascin-C (TNC), an extracellular matrix glycoprotein involved in HSC activation and migration, might be a specific target of miR-335 (16). Additionally, Murakami et al., in a CCL4-induced mouse liver fibrosis model, and also the human liver biopsy specimens, showed that overexpression of the miR-199 is tightly related to the progression of liver fibrosis in both mouse and human (17). In another study, Ji et al. found that overexpressed miR-27a induces hepatic stellate cell activation, an initial event in pathogenesis of liver fibrosis at least in partial, via targeting of retinoid X receptor alpha (18). This data suggests that chronic liver diseases can result in divergent microRNA expression patterns and alteration of microRNA gene expressions can affect chronic liver diseases such as liver fibrosis.

In conclusion, our results showed that suppression of TGF-β/smad signaling pathway, in addition to downregulation of the down-stream fibrotic genes of TGF-β1/smad signaling pathway but also via modulation of effective microRNA expressions on the pathogenesis of liver fibrosis, may have desirable therapeutic effects.
